# St. Louis Meeting American Medical Association

**Published:** 1886-05

**Authors:** 


### THE ST. LOUIS MEETING OF THE AMERICAN MEDICAL ASSOCIATION.

The thirty-seventh annual convention of the National Medical Association, which has been looked forward to so long, with hopes and fears, as being destined to mark a crisis in the history of the organization, and an important era in American medicine; and which has just closed its session at St. Louis, was a grand success—scientifically as well as socially. It is thought to have been the largest and most successful meeting of the Association ever held, not excepting the great St. Paul meeting, where the issue was made, and negatived, as to the eligibility of new-coders to seats as delegates in this body. There were about two thousand physicians present, and many of their ladies. Everything passed off smoothly and pleasantly. "All was harmony and smiles," says the *New York Record*.

The arrangements for the comfort and pleasure of guests were on a grand scale of magnificence; and the social feature of the great meeting is said to have eclipsed anything of the kind ever before attempted. The halls were transformed, for the time, into forests of tropical plants, brightened and perfumed with a wealth of roses and other flowers; while the atmosphere was cooled and made more fragrant by the perfumed spray of playing fountains, in which the festive goldfishes flashed in sport, (copyrighted). At night, the festal scenes were of oriental splendor; myriads of colored lights,—the dazzling toilettes of the ladies—the bright badges of members—gay uniforms of the brass-button brigade—the profusion of flowers;—the whole enlivened by strains of sweetest music (Dr. Merryman suggests "soul-stirring") awakened memories of the "feast of roses" —and conjured up visions of the Vale of Avoca.

As full reports of the scientific work—together with the full text of all the reports, addresses and other papers, will appear in the *Association Journal;* and as full accounts of the social entertainment have already been given in the secular press, we will, at this time, confine ourselves to such items of news as are of most interest, and which our readers are most anxious to learn.

Dr. E. H. Gregory, of St. Louis, was elected President, defeating Dr. W. W. Dawson, of Cincinnati, Ohio, two votes.

Dr. E. H. Miller, of Michigan, 1st; Dr. W. B. Welsh, of Arkansas, 2nd; Dr. W. H. Pancoast, of Pennsylvania, 3rd; and Dr. W. C. Wile, of *The New England Medical Monthly*, of Connecticut, 4th Vice President.

Chicago was chosen as the place of next meeting,—defeating Galveston by one vote,—and the first week in June the time.

The election of officers of sections was taken out of the hands of the nominating committee and given to the sections themselves—the argument being that the sections knew best who were the best qualified in each special branch for their positions. This has given rise to some dissatisfaction. Under the operation of this rule, a female physician was elected to the chairman(?)ship of the section of diseases of women and children, an honor never before conferred by the association on a woman. "Dr." Mary H. Thompson, of Chicago, was elected to the chairmanship of this section, over Professor Delaskie Miller, the distinguished chairman of the corresponding section in the Ninth International Medical Congress. We are pleased to say, however, that Mrs. Thompson, seeing the evident disfavor with which her election was received, had the good taste to resign. Dr. Miller was then elected.

The international congress question gave rise to no trouble. The report of the enlarged committee was received without opposition. The bolters seemed to "cave" entirely, when it was announced that the packed delegation, led by Agnew, Roberts & Co., of the Philadelphia County Medical Society, would not be admitted. So it was "all wind" at last, on the part of the "disgruntled." It is a fixed fact, then, that the International Medical Congress will be held, "as now proposed to be organized." Neither delegation from the Philadelphia

County Medical Society, was admitted; but as Shoemaker's party had been sent as delegates from the State Association, the code-loving, right thinking, and honest element of said society was represented after all. Trust Shoemaker to get there every time.

The only unpleasantness which occurred, was the emphatic flattening out of John B. Roberts, by the convention· He was sub-chairman of the Philadelphia packed delegation, and when refused admission, he offered a resolution of censure against Secretary Atkinson, the most popular man in the body. It was laid on the table unanimously, and J. B., then with an air of injured innocence, tendered his resignation as secretary to the section of surgery. The resignation was received with applause. The above is but a repetition of the old dog Tray story.

Texas was largely and well represented. Dr. Cupples was chairman of the delegation, Dr. Paine, of Galveston, was on the nominating committee; Dr. McLaughlin, on that of necrology; and Dr. C. H. Wilkinson was appointed a member of the section on state medicine.

Dr. N. S. Davis was made president of the Ninth International Medical Congress, vice Dr. Flint, deceased, and Surgeon-general J. B. Hamilton, M. H. S., was made secretary-general,—good!

The officers of sections are as follows :

Obstetrics—Dr. F. M. Johnson, Kansas City, chairman; Dr. W. B. Lawrence, Arkansas, secretary.

Surgery and Anotamy—Dr. H. H. Mudd, St. Louis, chairman; J. B. Roberts, (resigned) Philadelphia, secretary.

Dental and Oral Surgery—Dr. J. S. Marshall, Chicago, chairman; Dr. E. S. Talbott, Chicago, secretary.

Ophthalmology—Dr. X. C. Scott, Cleveland, Ohio, chairman; Dr. J. H. Thompson, Kansas City, secretary.

State Medicine—Dr. George H. Rohe, Maine, chairman; Surgeon M. H. S. Wyman, St Louis, Secretary.

Practice Medicine and Materia Medica—Dr. J. S. Lynch, Baltimore, chairman; Dr. J. B. Merwin, Louisville, secretary.

Medical Jurisprudence—Dr. I. N. Quimby, of New Jersey, chairman.

It is proposed to create a section on Dermatology and Syphilis, at the next meeting.

Galveston would have been chosen as the next place of meeting, but for financial considerations. It was urged that it takes money to run the JOURNAL. If the association were to meet so far off, on the edge of the continent, the attendance would be slim, ergo, the receipts small. Will such argument always work an injury and injustice to the south?

